# Employing urinary biomarkers to infer the absence of acute kidney disease in outpatients with a single serum creatinine measurement

**DOI:** 10.1080/0886022X.2024.2427161

**Published:** 2024-11-26

**Authors:** Shih-Ping Hsu, Chiang-Ting Chien

**Affiliations:** a Department of Internal Medicine, Far Eastern Memorial Hospital, New Taipei, Taiwan; b School of Life Science, National Taiwan Normal University, Taipei, Taiwan; c College of Medicine, National Taiwan University, Taipei, Taiwan; d General Education Center, Lunghwa University of Science and Technology, Taoyuan, Taiwan

**Keywords:** Acute kidney disease, acute renal dysfunction, ASSESS-AKI, spot urine creatinine-to-osmolality ratio, neutrophil gelatinase-associated lipocalin

## Abstract

**Introduction:**

In outpatient settings, it is challenging to exclude acute kidney disease (AKD) based on a single serum creatinine (SCr) measurement. This retrospective study aimed to explore the usefulness of urinary biomarkers and a novel functional biomarker, a spot urine creatinine-to-osmolality ratio (sUCr/Osm), for inferring the absence of AKD.

**Methods:**

The cohort was from the ASSESS-AKI Study. ‘No AKD’ was defined as the absence of a SCr increase ≥ 26.5 μmol/L between the preceding visit and the index visit, with a three-month interval. Urinary neutrophil gelatinase-associated lipocalin (UNGAL) was selected as the representative biomarker out of six known candidates. UNGAL levels < 100 ng/mL indicated a positive test. sUCr/Osm values ≥ 7.07 indicated a positive test.

**Results:**

Of the 1,570 participants, 38.0% were female. The mean age (mean ± SD) was 64.6 ± 13.0 years, and the mean SCr level was 102.5 ± 51.4 μmol/L. The area under the receiver operating characteristic curve for UNGAL in identifying ‘No AKD’ for all participants was 0.548 (95% confidence interval: 0.495–0.600), whereas that for sUCr/Osm was 0.578 (0.525–0.630). The sensitivity of UNGAL was 0.867 (0.849–0.884), with a positive predictive value of 0.917 (0.902–0.932) and an accuracy of 0.808 (0.788–0.827). The corresponding values of sUCr/Osm were 0.926 (0.912–0.939), 0.906 (0.891–0.921), and 0.845 (0.827–0.863). In individuals whose SCr-derived estimated glomerular filtration rate was < 60 mL/min/1.73 m^2^, sUCr/Osm performed comparably to UNGAL.

**Conclusion:**

Using sUCr/Osm to infer the absence of AKD in outpatients with a single SCr measurement may be as effective as using UNGAL.

## Introduction

Outpatient renal care relies on an understanding of the stability of renal function rather than a single measurement of the serum creatinine (SCr) level or an estimated glomerular filtration rate (eGFR). Markedly elevated SCr levels of uncertain duration may necessitate referral to the emergency department or hospitalization [1, 2]. Urinary biomarkers may facilitate the exclusion of acute kidney dysfunction in a timely manner when only one SCr measurement is available. However, this approach has not been explored in outpatient settings.

While acute kidney injury (AKI) is defined as an increase in SCr ≥ 26.5 μmol/L within 48 h [3], acute kidney disease (AKD) is defined as the persistent loss of kidney function over 7–90 days [4]. Chronic kidney disease (CKD) is diagnosed when impaired renal function remains stable without AKI or AKD for at least three months [5]. Therefore, ‘No AKD’ is defined as the absence of a documented increase in SCr ≥ 26.5 μmol/L during the three months prior to an index outpatient visit.

To investigate elevated SCr levels of uncertain duration in outpatient settings, one spot urine sample is more timely than closely tracking at least two-day changes in SCr levels or six-hour urine output (UO). While routine dipstick and microscopic analyses may suggest pathological kidney damage, they lack standardized criteria and relevance to chronicity [6]. Urinary indices, such as the fractional excretion of urea or sodium, may also be ineffective in assessing renal function stability [7]. Several urinary AKI biomarkers, including urinary neutrophil gelatinase-associated lipocalin (UNGAL) [8], have been proposed for the prediction of the acute deterioration of renal function [9–11]. However, there is inadequate evidence to support the effectiveness of performing urinary biomarker analysis in outpatients who have no known kidney injuries or exposures [9]. Additionally, the cost and availability of these biomarker tests limit their widespread use [10].

In contrast, the spot urine creatinine-to-osmolality ratio (sUCr/Osm) is an economical and globally-available biomarker that indicates the relative excretion rate of urine creatinine (UCr) to osmoles, irrespective of water intake, urine volume, the intervoid interval, and underlying CKD status [12]. The rationale for this biomarker of momentary excretory function is based on the physiological presumption that the excretion amounts of creatinine and osmoles in urine are stable and predictable in individuals with stable metabolic conditions [13, 14]. To maintain homeostasis, the body must constantly excrete creatinine and osmoles through adaptable urine volumes. A spot urine sample is one of four to six urinations per day [15]. In addition, the urinary excretions of creatinine and osmoles are proportional without pulsatile variations after meals [16]. Therefore, some indicators based on spot UCr or urine osmolality (UOsm) have long been used in the clinic [17, 18].

The objective of this study was to explore the efficacy of known urinary biomarker tests and the sUCr/Osm tests performed at an index outpatient visit for the inference of the absence of AKD during the preceding three months. This study also presents a flowchart developed to guide outpatient renal care on the basis of a single SCr-derived eGFR and an accompanying sUCr/Osm value.

## Materials and methods

### Study population

The cohort for the current study was derived from the Assessment, Serial Evaluation, and Subsequent Sequelae of Acute Kidney Injury (ASSESS-AKI) study. The original datasets were provided by the National Institute of Diabetes and Digestive and Kidney Diseases (NIDDK) Central Repository upon request.

The aim of the ASSESS-AKI study was to investigate the impact of an AKI episode during hospitalization on the future occurrence and progression of CKD. Participants provided written informed consent, and institutional review boards at participating institutions granted approval [19, 20]. The detailed project information has been published [19, 20]. This cohort study enrolled individuals aged 18–89 years who were hospitalized between December 2009 and February 2015. Subsequent outpatient visits and the collection of blood and urine samples were scheduled at three and 12 months postdischarge and at other intervals thereafter.

**Figure 1.**
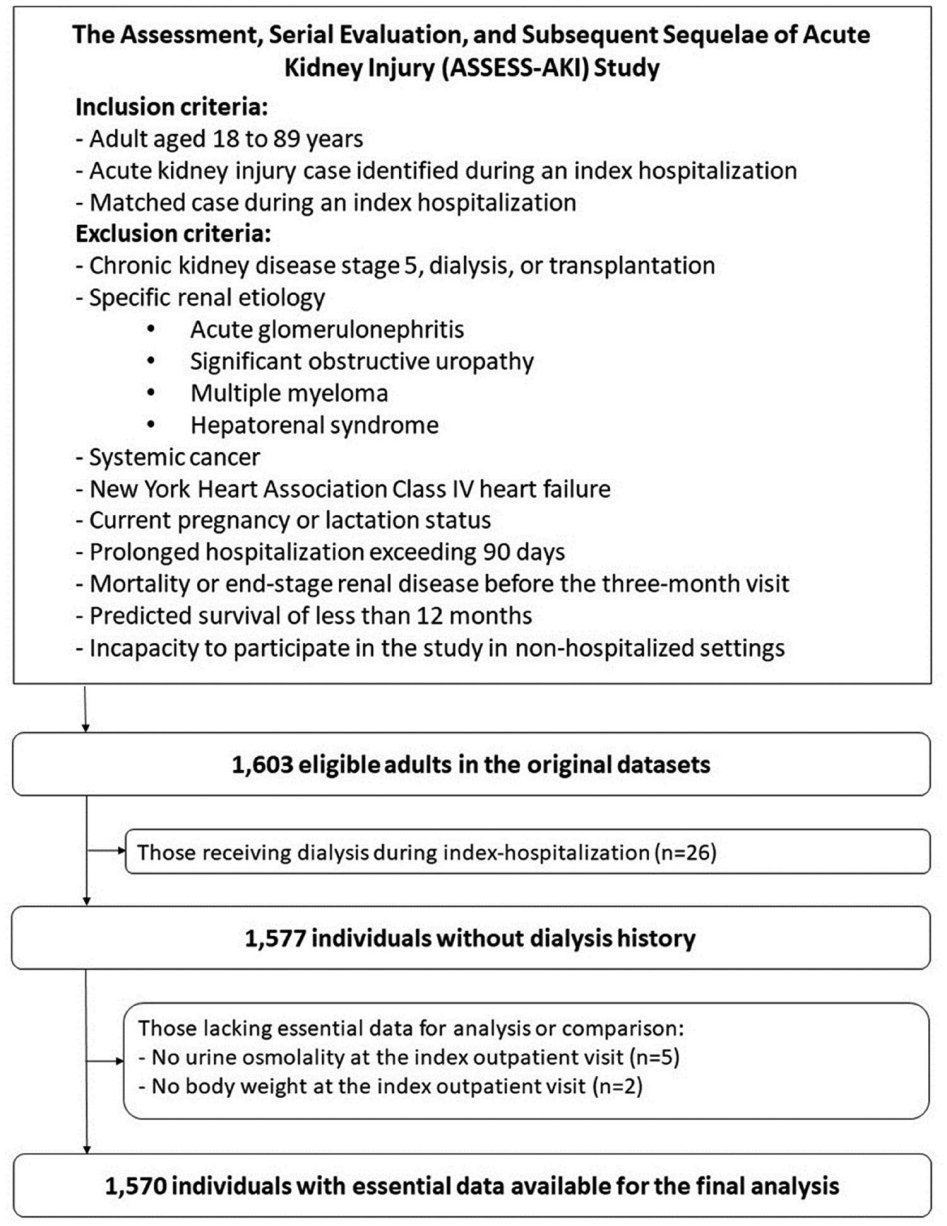
The sampling flow chart.

The original study included data from 1,603 adults. After 26 individuals with a history of in-hospital dialysis and seven with missing essential data were excluded, the final dataset for this analysis included data from 1,570 individuals. Figure 1 shows the sampling flowchart. The current study protocol was reviewed and granted an exemption from requiring ethics approval by the Research Ethics Review Committee, Far Eastern Memorial Hospital (approval number 111176-W). Patient consent was waived as this study was based on publicly available data. This study was conducted in accordance with the Declaration of Helsinki.

### Participant characteristics

During the index hospitalization enrollment, participants provided their date of birth, sex, and race. The diagnosis and severity assessment of AKI were based on *a* ≥ 50% relative increase or ≥ 26.5 μmol/L absolute increase in peak SCr in inpatients compared with a well-established baseline SCr prior to hospitalization. Due to limitations in data collection quality, the UO criteria for AKI were not applied. Preexisting CKD was defined as a baseline SCr-derived 2009 CKD-EPI eGFR value of < 60 mL/min/1.73 m^2^. Other comorbidities were identified using medical records or *via* self-reports [19, 20].

At the index outpatient visit three months after discharge, the participants’ height and body weight were either self-reported or measured. The participants were instructed to bring all prescriptions and over-the-counter medications taken within the past 30 days. If the participants did not bring their medications, the necessary information was obtained *via* interview.

### Blood and urine tests

Sample collection did not require fasting. The Central Lab at the University of Minnesota analyzed the blood and urine samples. The SCr levels were determined *via* an IDMS-traceable assay. The local laboratory measured the SCr levels at the outpatient visits.

### Urinary biomarkers

Six urinary biomarkers for AKI were tested, including NGAL, kidney injury molecule-1 (KIM-1), interleukin-18 (IL-18), monocyte chemoattractant protein 1 (MCP-1), uromodulin (UMOD), and chitinase 3-like 1 (YKL-40) [21]. The samples were refrigerated if not processed within 30 min of collection. All samples were processed within six hours after collection, beginning with centrifugation at 1000 × g for 10 min at room temperature. After centrifugation, the samples were aliquoted, frozen, and stored at −70 °C until measurement. The assays used for these measurements have been published previously [22].

### Outpatient settings

The test performance was evaluated in all enrolled individuals and in the subgroup with a low SCr-derived eGFR (< 60 mL/min/1.73 m^2^ using the race-neutral 2021 CKD-EPI equation). These scenarios correspond to the challenging situations for outpatients who lack paired SCr data for comparison, especially when the available eGFR is low.

### ‘No AKD’ vs. ‘AKD’

‘No AKD’ was defined as the absence of a documented interval increase in SCr ≥ 26.5 μmol/L over the three months since discharge. In individuals who experienced AKI during hospitalization, this definition signifies the absence of new, superimposed AKD following discharge. Conversely, the condition of a larger increase was defined as ‘AKD’. These definitions are more compatible with prudent renal care than those based on increased folds of SCr levels.

### Candidate tests

The areas under the receiver operating characteristic (ROC) curves (AUCs) of six urinary biomarkers were compared to identify one biomarker as a representative test for the general outpatient setting. The cutoff value for this biomarker was determined using the maximum Youden index method.

Both sUCr/Osm and a derived sUCr/Osm index, which was indexed by personalized estimated sUCr/Osm [12], were tested for their efficacy in inferring renal function stability. Additionally, a race-neutral sUCr/Osm index, which was indexed by the personalized estimated sUCr/Osm *via* a race-neutral, modified equation [0.340 × (age in year)^−0.070^ × (BW in kg)^0.283^ × (BUN in mmol/L)^−0.356^ × (SCr in μmol/L)^0.754^[unpublished], was also examined.

In this exploratory study, the cutoffs of the sUCr/Osm tests were tentatively set at the lower limits of the left-sided reference intervals [12], above which the values of 95% of ordinary adults are typically observed. Accordingly, a sUCr/Osm value ≥ 7.07 when UCr was reported in μmol/L was considered positive, and smaller values were considered negative. A value ≥ 0.50 for the two derived indices was defined as positive, and smaller values were considered negative.

### Statistical analysis

The ASSESS-AKI study datasets were obtained in SAS files. To merge the relevant datasets, each participant’s SUBJ_ID was utilized as the identifier. IBM^®^ SPSS^®^ Statistics version 22.0 (New York, USA) was used in the current analysis.

Unless otherwise specified, the data are presented as mean ± standard deviation, median [interquartile range], or number (%). If applicable, the differences were analyzed using Student’s t test, the chi-square test, or one-way analysis of variance (ANOVA) with the Bonferroni method as the *post hoc* test. Factors associated with ‘No AKD’ were examined *via* multivariable logistic regression. A *P* value < 0.05 was considered statistically significant for two-tailed tests, unless otherwise stated.

## Results

### Participant characteristics

The mean age of the 1,570 participants in this study was 64.6 ± 13.0 years. Of them, 38.0% were female, and 12.6% were black. The mean body mass index (BMI) was 30.1 ± 7.7 kg/m^2^. Hypertension was present in 72.8% of the participants, and 41.0% of the participants had diabetes mellitus. Nearly half (48.5%) of the participants were taking angiotensin converting enzyme inhibitors or angiotensin II receptor blockers, and 42.7% were taking diuretics. The use of sodium glucose cotransporter 2 inhibitors was not reported, despite their FDA approval in the US in January 2014. During the index hospitalization, 47.3% of the patients experienced AKI.

At the index outpatient visit, 1,424 participants (90.7%) were identified as having ‘No AKD’ and 146 (9.3%) were identified as having ‘AKD’. The median interval between the preceding SCr measurement (the last in-hospital measurement) and the index visit was 90 days [74–106 days].

Table 1 presents a comprehensive overview of the participant characteristics in each group.

**Table 1.** Participant characteristics and laboratory data at the index outpatient visit and comparisons between the groups with and without acute kidney disease.

	All individuals	No AKD	AKD	*P* *
	*N* = 1570	*N* = 1424	*N* = 146	
Age, year	64.6 ± 13.0	64.5 ± 12.9	66.3 ± 13.1	0.11
Female sex	596 (38.0)	557 (39.1)	39 (26.7)	**0.003**
Body weight, kg	90.3 ± 23.5	90.5 ± 23.5	87.9 ± 23.8	0.20
BMI, kg/m^2^	30.1 ± 7.7	31.1 ± 7.7	29.6 ± 7.3	**0.02**
Race				0.50
Asian	30 (1.9)	26 (1.8)	4 (2.7)	
Black	198 (12.6)	181 (12.7)	17 (11.6)	
White	1286 (81.9)	1169 (82.1)	117 (80.1)	
Other Races	56 (3.6)**	48 (3.4)	8 (5.6)	
Smoker	691 (44.0)	619 (43.5)	72 (49.3)	0.18
Comorbidity				
CKD	605 (38.5)	507 (35.6)	98 (67.1)	**<0.001**
Hypertension	1143 (72.8)	1029 (72.3)	114 (78.1)	0.30
Diabetes mellitus	643 (41.0)	576 (40.4)	67 (45.9)	0.20
CAD	686 (43.7)	606 (42.6)	80 (54.8)	**0.02**
CHF	323 (20.6)	282 (19.8)	41 (28.1)	0.06
Chronic lung Dis	342 (21.8)	312 (21.9)	30 (20.5)	0.71
Chronic liver Dis	61 (3.9)	56 (3.9)	5 (3.4)	0.91
Lupus	15 (1.0)	14 (1.0)	1 (0.7)	0.90
In-hospital AKI				
Any stage	742 (47.3)	664 (46.6)	78 (53.4)	0.12
Stage 1	552 (35.2)	486 (34.1)	66 (45.2)	**0.04**
Stage 2	119 (7.6)	110 (7.7)	9 (6.2)	
Stage 3	71 (4.5)	68 (4.8)	3 (2.1)	
preexisting CKD	297 (18.9)	248 (17.4)	49 (33.6)	**<0.001**
Medication use				
ACEI	514 (32.7)	461 (32.4)	53 (36.3)	0.34
ARB	248 (15.8)	222 (15.6)	26 (17.8)	0.48
β-adrenergic blocker	892 (56.8)	791 (55.5)	101 (69.2)	**0.002**
CCB	353 (22.5)	310 (21.8)	43 (29.5)	**0.03**
Corticosteroids	276 (17.6)	251 (17.6)	25 (17.1)	0.88
NSAID	80 (5.1)	75 (5.3)	5 (3.4)	0.34
Diuretics	671 (42.7)	579 (40.7)	92 (63.0)	**<0.001**
Loop	469 (29.9)	397 (27.9)	72 (49.3)	**<0.001**
Thiazides	243 (15.5)	214 (15.0)	29 (19.9)	0.12
ARA	120 (7.6)	101 (7.1)	19 (13.0)	**0.01**
Blood biochemistry				
Urea Nitrogen, mmol/L	8.08 ± 4.51	7.58 ± 3.79	13.06 ± 7.16	**<0.001**
Creatinine, μmol/L	102.5 ± 51.4	96.1 ± 41.0	167.8 ± 86.6	**<0.001**
2009 CKD-EPI Cr eGFR, ml/min/1.73 m^2^	70.1 ± 25.9	73.1 ± 24.8	41.5 ± 18.3	**<0.001**
2021 CKD-EPI Cr eGFR, ml/min/1.73 m^2^	72.5 ± 25.7	75.5 ± 24.4	43.6 ± 19.2	**<0.001**
Random glucose, mmol/L	7.1 ± 3.9	7.1 ± 3.9	7.2 ± 3.9	0.656
Urine biochemistry				
Creatinine, μmol/L	8826 ± 6444	8841 ± 6510	8685 ± 5782	0.781
Osm, mOsm/Kg	512 ± 211	519 ± 214	446 ± 158	**<0.001**
UCr/Osm	14.54 [10.99–19.81]	14.39 [10.93–19.58]	16.63 [11.54–24.65]	**0.002**
UACR, mg/mmol	1.5 [0.7–6.1]	1.5 [0.7–5.8]	2.4 [1.0–9.2]	0.092
Urinary biomarkers				
NGAL, ng/mL	23 [11–56]	23 [11–53]	26 [10–92]	**0.007**
KIM-1, pg/mL	1298 [502–2820]	1281 [462–2751]	1416 [682–3661]	**0.026**
IL-18, pg/mL	25 [13–49]	25 [13–49]	21 [12–48]	0.746
MCP-1, pg/mL	236 [98–495]	234 [96–484]	243 [120–730]	0.134
UMOD, ng/mL	2598 [1563–4067]	2634 [1619–4101]	2029 [1126–3297]	**0.015**
YKL-40, pg/mL	486 [205–1100]	484 [205–1059]	513 [203–1666]	0.067

Note: Data are presented as mean ± standard deviation or number (%). Bold values represents statistical significance.

*Comparisons between the groups of ‘No AKD’ and ‘AKD’ using the Student’s t-test, chi-square test, or Fisher’s exact test as appropriate.

**Included are 15 American Indian or Alaskan Native, 10 Native Hawaiian or other Pacific Islander, and 31 more than one race.

Abbreviations: ACEI, angiotensin converting enzyme inhibitor; AKI, acute kidney injury; AKD, acute kidney disease; ARA, aldosterone receptor antagonist; ARB, angiotensin II receptor blocker; BMI, body mass index; CAD, coronary artery disease; CCB, calcium channel blocker; CHF, chronic heart failure; CKD, chronic kidney disease; Cr, creatinine; eGFR, estimated glomerular filtration rate; Dis, disease; L-18, interleukin-18; K, potassium; KIM-1, kidney injury molecule-1; MCP-1, monocyte chemoattractant protein 1; Na, sodium; NGAL, neutrophil gelatinase-associated lipocalin; NSAID, non-steroidal anti-inflammatory drug; Osm, osmolality; UCr/Osm, urine creatinine-to-osmolality ratio; UACR, urine albumin to creatinine ratio; UMOD, uromodulin; YKL-40, chitinase 3-like 1.

To convert creatinine to mg/dL, divided by 88.4; cystatin C to mg/dL, 74.9; glucose to mg/dL, 0.0555; UACR to mg/g, 0.113; and urea nitrogen to mg/dL, 0.357.

### Blood and urine tests

At the index outpatient visit, the mean SCr level was 102.5 ± 51.4 μmol/L, and the mean eGFR according to the 2021 race-neutral CKD-EPI equation was 72.5 ± 25.7 mL/min/1.73 m^2^. The mean sUCr/Osm in the spot-urine samples was 16.37 ± 8.33, and the UNGAL concentration was 83 ± 241 ng/mL. These results are presented in Table 1.

### Factors associated with ‘no AKD’

A forward conditional, logistic regression analysis was conducted to further investigate the relationship between the condition of ‘No AKD’ and variables showing significant between-group differences, as shown in Table 1. This analysis considered conventional factors, such as age, sex, the presence of in-hospital AKI, and the presence of preexisting CKD, as fixed covariables for adjustment. Considering the collinearity among the renal function parameters, SCr was designated as the representative variable. Among the six urinary biomarkers, UNGAL was selected as the representative biomarker due to its small *P* value. The final model revealed that only SCr and BMI were significant covariables associated with the condition of ‘No AKD’ (Nagelkerke R Squared = 0.242, *P* < 0.001). SCr had a negative association (unstandardized coefficient B = −1.692, *P* < 0.001), and BMI had a positive association (*B* = 0.034, *P* = 0.024).

### Test performance in the general outpatient setting

**Figure 2.**
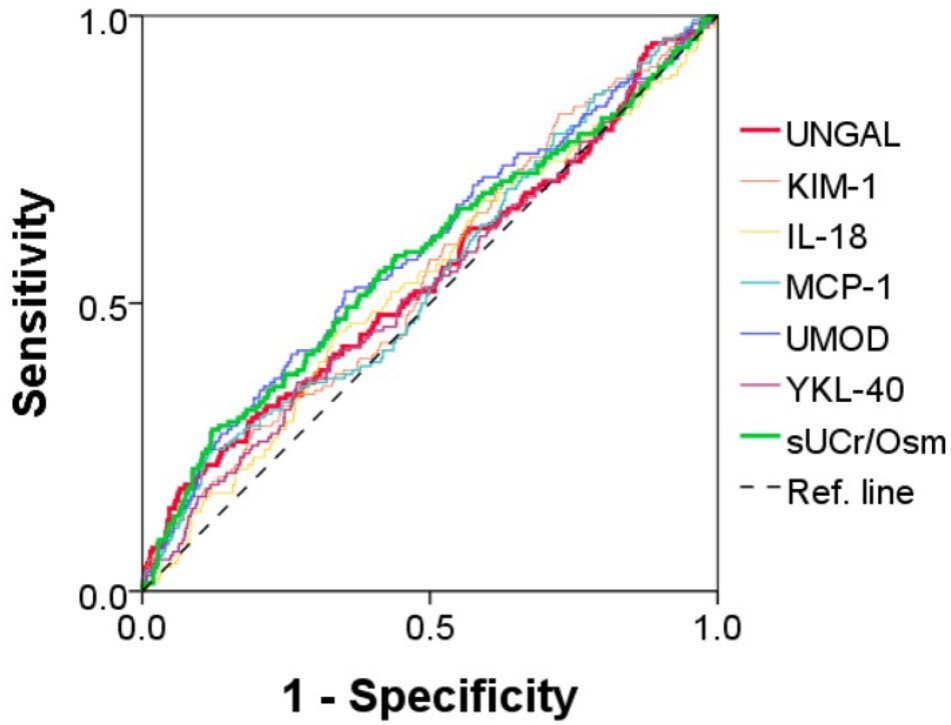
The areas under the ROC curves of the urinary biomarkers. UNGAL, urinary neutrophil gelatinase-associated lipocalin; KIM-1, kidney injury molecule-1; IL-18, interleukin-18; MCP-1, monocyte chemoattractant protein 1; UMOD, uromodulin; YKL-40, chitinase 3-like 1; sUCr/Osm, spot urine creatinine-to-osmolality ratio.

Among the 1,570 participants, the AUCs of the six urinary biomarkers for differentiating between ‘No AKD’ and ‘AKD’ were as follows: UNGAL, 0.548 (95% confidence interval (95% CI): 0.495–0.600); KIM-1, 0.551 (95% CI: 0.503–0.600); IL-18, 0.537 (95% CI: 0.487–0.588); MCP-1, 0.548 (95% CI: 0.498–0.598); UMOD, 0.588 (95% CI: 0.537–0.639); and YKL-40, 0.531 (95% CI: 0.477–0.586). The AUC of sUCr/Osm was comparable at 0.578 (95% CI: 0.525–0.630) (Figure 2).

As the AUCs of the six candidate biomarkers were not significantly different and a semiquantitative dipstick test of UNGAL had been performed in the emergency department [11], UNGAL was selected as the representative reference biomarker for further comparison with sUCr/Osm. For inferring ‘No AKD’, UNGAL levels < 100 ng/mL were classified as positive.

As shown in Table 2, in the general outpatient setting, with a prevalence of 90.7%, the sensitivity of the UNGAL test for inferring ‘No AKD’ was 0.867 (95% CI: 0.849–0.884), which was significantly lower than that of the sUCr/Osm test. In contrast, the UNGAL test had a higher positive predictive value (PPV) (0.917; 95% CI: 0.902–0.932), though it was not significantly superior to that of the sUCr/Osm test. The UNGAL test yielded an accuracy of 0.808 (95% CI: 0.788–0.827), and the sUCr/Osm test produced higher values.

**Table 2.** Test performance metrics evaluated in two groups: (A) all enrolled outpatients and (B) outpatients with eGFR < 60 mL/min/1.73 m^2^.

(A) All enrolled outpatients				
		No AKD	AKD	Total
Test		*n* = 1,424	*n* = 146	*n* = 1,570
UNGAL	(+)	1234	112	1346
	(-)	190	34	224
sUCr/Osm	(+)	1318	137	1455
	(-)	106	9	115
Index 1	(+)	1309	126	1435
	(-)	115	20	135
Index 2	(+)	1310	125	1435
	(-)	114	21	135
	UNGAL	sUCr/Osm	Index 1	Index 2
Sensitivity (95% CI)	0.867 (0.849–0.884)	0.926 (0.912–0.939)	0.919 (0.905–0.933)	0.920 (0.906–0.934)
Specificity (95% CI)	0.233 (0.164–0.301)	0.062 (0.023–0.101)	0.137 (0.081–0.193)	0.144 (0.087–0.201)
PPV (95% CI)	0.917 (0.902–0.932)	0.906 (0.891–0.921)	0.912 (0.898–0.927)	0.913 (0.898–0.927)
NPV (95% CI)	0.152 (0.105–0.199)	0.078 (0.029–0.127)	0.148 (0.088–0.208)	0.156 (0.094–0.217)
LR+ (95% CI)	1.130 (1.031–1.238)	0.986 (0.944–1.031)	1.065 (0.997–1.138)	1.074 (1.004–1.150)
LR- (95% CI)	0.573 (0.419–0.791)	1.208 (0.625–2.334)	0.586 (0.378–0.918)	0.557 (0.361–0.858)
Accuracy (95% CI)	0.808 (0.788–0.827)	0.845 (0.827–0.863)	0.846 (0.829–0.864)	0.848 (0.830–0.866)
Youden index	0.099	−0.013	0.056	0.064
(B) Outpatients with eGFR < 60 ml/min/1.73 m²				
		No AKD	AKD	Total
Test		*n* = 400	*n* = 121	*n* = 521
UNGAL	(+)	319	91	410
	(-)	81	30	111
sUCr/Osm	(+)	369	112	481
	(-)	31	9	40
Index 1	(+)	342	101	443
	(-)	58	20	78
Index 2	(+)	345	100	445
	(-)	55	21	76
	UNGAL	sUCr/Osm	Index 1	Index 2
Sensitivity (95% CI)	0.798 (0.758–0.837)	0.923 (0.896–0.949)	0.855 (0.820–0.890)	0.863 (0.829–0.896)
Specificity (95% CI)	0.248 (0.171–0.325)	0.074 (0.028–0.121)	0.165 (0.099–0.231)	0.174 (0.106–0.241)
PPV (95% CI)	0.778 (0.738–0.818)	0.767 (0.729–0.805)	0.772 (0.733–0.811)	0.775 (0.736–0.814)
NPV (95% CI)	0.270 (0.188–0.353)	0.225 (0.094–0.354)	0.256 (0.160–0.353)	0.276 (0.176–0.377)
LR+ (95% CI)	1.060 (0.947–1.188)	0.997 (0.941–1.056)	1.024 (0.937–1.120)	1.044 (0.953–1.143)
LR- (95% CI)	0.817 (0.566–1.178)	1.042 (0.510–2.127)	0.877 (0.551–1.398)	0.792 (0.500–1.255)
Accuracy (95% CI)	0.670 (0.629–0.710)	0.726 (0.687–0.764)	0.695 (0.655–0.734)	0.702 (0.663–0.742)
Youden index	0.045	−0.003	0.020	0.036

Note: Cutoffs for the tests: For UNGAL: < 100 ng/mL, (+); otherwise, (-). For sUCr/Osm: ≥ 7.07 when Cr in μmol/L, (+); otherwise, (-). For Index 1 or 2: ≥ 0.5, (+); otherwise, (-).

Abbreviations: CI, confidence interval; eGFR, estimated glomerular filtration rate calculated using the race-neutral 2021 CKD-EPI creatinine equation; Index 1, the sUCr/Osm Index, sUCr/Osm divided by personalized estimated sUCr/Osm [12]; Index 2, the race-neutral sUCr/Osm Index, sUCr/Osm divided by race-neutral, personalized estimated sUCr/Osm [12]; LR-, negative likelihood ratio; LR+, positive likelihood ratio; NPV, negative predictive value; PPV, positive predictive value; sUCr/Osm, spot urine creatinine-to-osmolality ratio; UNGAL, urinary neutrophil gelatinase-associated lipocalin.

### Test performance in outpatients with eGFRs < 60 mL/min/1.73 m^2^

Among the 1,570 participants, 1,049 had an eGFR ≥ 60 mL/min/1.73 m^2^, including 97.6% with ‘No AKD’. Among the remaining 521 participants with an eGFR < 60 mL/min/1.73 m^2^, 76.8% had ‘No AKD’.

As shown in Table 2, among the 521 outpatients with an eGFR < 60 mL/min/1.73 m^2^, the UNGAL test demonstrated a sensitivity of 0.798 (95% CI: 0.758–0.837) for inferring ‘No AKD’, which was lower than that of the sUCr/Osm test. Although the UNGAL test had a PPV of 0.778 (95% CI: 0.738–0.818), it was not significantly superior to that of the sUCr/Osm test. The UNGAL test had an accuracy of 0.670 (95% CI: 0.629–0.710), and the sUCr/Osm test demonstrated higher values.

## Discussion

The results of this exploratory study indicate that the UNGAL test may be an effective tool for inferring ‘No AKD’ in terms of sensitivity, PPV, and accuracy in the general outpatient setting or in individuals with an eGFR < 60 mL/min/1.73m^2^. In addition, the sUCr/Osm test demonstrated comparable efficacy to the UNGAL test. Based on the comparable performances of the original sUCr/Osm test and its derived indices and the simplicity, this test is more convenient for clinical use and was selected as the representative biomarker in this study.

For outpatients, a high office blood pressure (BP) measurement may indicate a new diagnosis or poor control of chronic hypertension [23]. In contrast, a normal value may suggest that an individual’s BP has been normal over an uncertain duration or well controlled following the last visit. Similar to office BP measurements, spot urine tests are convenient and informative tools in outpatient settings. While a single SCr value indicates the current level of renal excretory function, regardless of whether it is acute or chronic, spot urine tests provide a snapshot of the renal condition since the last voiding. If no abnormalities are observed on spot urine tests, it can be inferred that the renal function has remained stable over an uncertain duration. While urinary AKI biomarkers, such as UNGAL, may suggest the presence of tissue injury, sUCr/Osm may indicate the adequacy of excretory function [12].

The use of UNGAL has been studied extensively in high-risk inpatients [9]. In an emergency room setting, a three-level colorimetric dipstick method for the semiquantitative determination of UNGAL based on immunoassays (NGAL gRAD dipsticks, Bioporto, Denmark) has also been tested [11]. The same ELISA method (NGAL ELISA Kit 036, Bioporto, Denmark) was used in the ASSESS-AKI study [22] and, therefore, in the current study. In the general outpatient setting, the current study revealed that the UNGAL test achieved a sensitivity of 0.867 and a PPV of 0.917 with a cutoff value of 100 ng/mL to infer ‘No AKD’. According to a report in which Bioporto’s serial products were used to measure UNGAL and determine AKI risk, a value > 300 ng/ml exhibited increased specificity for the detection of AKI [24]. In the general outpatient setting of the current study, with a cutoff value of 300 ng/ml, the sensitivity increased to 0.952 and the PPV increased to 0.916. However the specificity decreased to 0.144, resulting in a lower Youden index value of 0.096.

In contrast, the performance of the sUCr/Osm test to infer ‘No AKD’ in outpatient settings has been evaluated for the first time in this study. In the general outpatient setting, when the prevalence of ‘No AKD’ was 90.7%, the sUCr/Osm test, with a cutoff preliminarily set at the lower limit of the left-sided reference interval, had a sensitivity of 0.926 and a PPV of 0.906 (Table 2). While the prevalence of ‘No AKD’ was lower at 76.8% in participants with an eGFR < 60 mL/min/1.73 m^2^, the sensitivity was 0.923 and the PPV was 0.767 (Table 2). Further analysis revealed that the prevalence was 97.6% in participants with an eGFR ≥ 60 mL/min/1.73 m^2^, with a sensitivity of 0.927 and a PPV of 0.974. The high sensitivity (approximately 0.93) and PPV values (close to the respective prevalence rates) indicate that the implementation of this convenient test will assist in reducing unnecessary tests, costs, and anxiety associated with extended observation periods, especially when elevated SCr levels are identified incidentally. These findings also corroborate the aforementioned result that a lower SCr (higher eGFR) is associated with a greater likelihood of ‘No AKD’.

To further investigate the positive correlation between BMI and the likelihood of having ‘No AKD’ in the current study, the participants were divided into three categories on the basis of their BMI: low (BMI < 18.5 kg/m^2^), moderate (BMI ≥ 18.5 and < 35.0 kg/m^2^), and high (BMI ≥ 35.0 kg/m^2^). The moderate group is consistent with the BMI distributions reported in previous studies that estimated renal excretory function using SCr [25, 26] and sUCr/Osm [12]. The prevalence rates of ‘No AKD’ for the three levels were 88.9% (16/18), 90.4% (1057/1169), and 91.6% (351/383), respectively. A larger body size or BMI may increase the rate of ‘No AKD’ in two ways when the absence of an absolute increase in SCr ≥ 26.5 μmol/L is used as a criterion. In addition to the disproportionate hyperfiltration associated with obesity [27], a larger body size necessitates a greater accumulation of creatinine for a given level of SCr elevation. Furthermore, the influence of BMI on the efficacy of the UNGAL and sUCr/Osm tests was evaluated. The UNGAL test demonstrated a sensitivity of 0.875 (14/16), 0.868 (917/1057), and 0.863 (303/351), respectively, for inferring ‘No AKD’ in the entire participant cohort across the three BMI categories. The sensitivities of the sUCr/Osm test were 1.0 (16/16), 0.933 (986/1057), and 0.900 (316/351), respectively, for the three BMI categories. Therefore, the efficacy of the sUCr/Osm test may be diminished in individuals with a high BMI (≥ 35.0 kg/m^2^), though it is more effective than the UNGAL test.

To elucidate the reason for the greater mean sUCr/Osm value in the ‘AKD’ group in this study, an additional analysis was conducted to examine the difference in the SCr values obtained three months before and nine months after the index visit. Among the 146 participants in the ‘AKD’ group, 115 had follow-up SCr measurements obtained nine months after the index visit. Among these 115 participants, 64 did not ultimately have an increase in SCr ≥ 26.5 μmol/L during the 12-month period. These findings indicate that in at least 43.8% of the 146 individuals with ‘AKD’, the increase in SCr observed at the index visit was temporary and reversible. The recovery from elevated SCr levels was most likely through higher-than-ordinary excretion rates of urinary Cr, presenting as higher SCr/Osm values than the mean of the ‘No AKD’ group. Consequently, it is reasonable to posit that the mean SUCr/Osm value in the ‘AKD’ group was paradoxically higher than that in the ‘No AKD’ group. This finding also suggests that a higher-than-average sUCr/Osm value in an individual with ‘AKD’ at the index visit may imply the potential for the reversal of increased SCr levels.

The limitations of this exploratory study are primarily due to a lack of information regarding the history and physical findings of the participants at the index visit. Although nearly half of the false-positive cases may be those with higher-than-average sUCr/Osm values and eventual reversal of SCr levels suggesting potential recovery from ‘AKD’, it is essential to rely on clinical information to exclude other conditions, including poor intake resulting in lower-than-­predicted osmolar loads, hypovolemia with a tendency to retain osmoles [28], and inadequate urine output to excrete both the expected creatinine and osmolar loads. Clinical clues can also assist in the exclusion of false-negative cases resulting from creatinine production below estimated amounts, excess osmolar loads due to hypercatabolism or uptake, or additional osmole excretion with diuretic usage or glucosuria. Notably, 5% of the participants consistently have sUCr/Osm values lower than the fifth percentile limit, as the cutoff is set at the lower limit of the left-sided reference interval.

**Figure 3.**
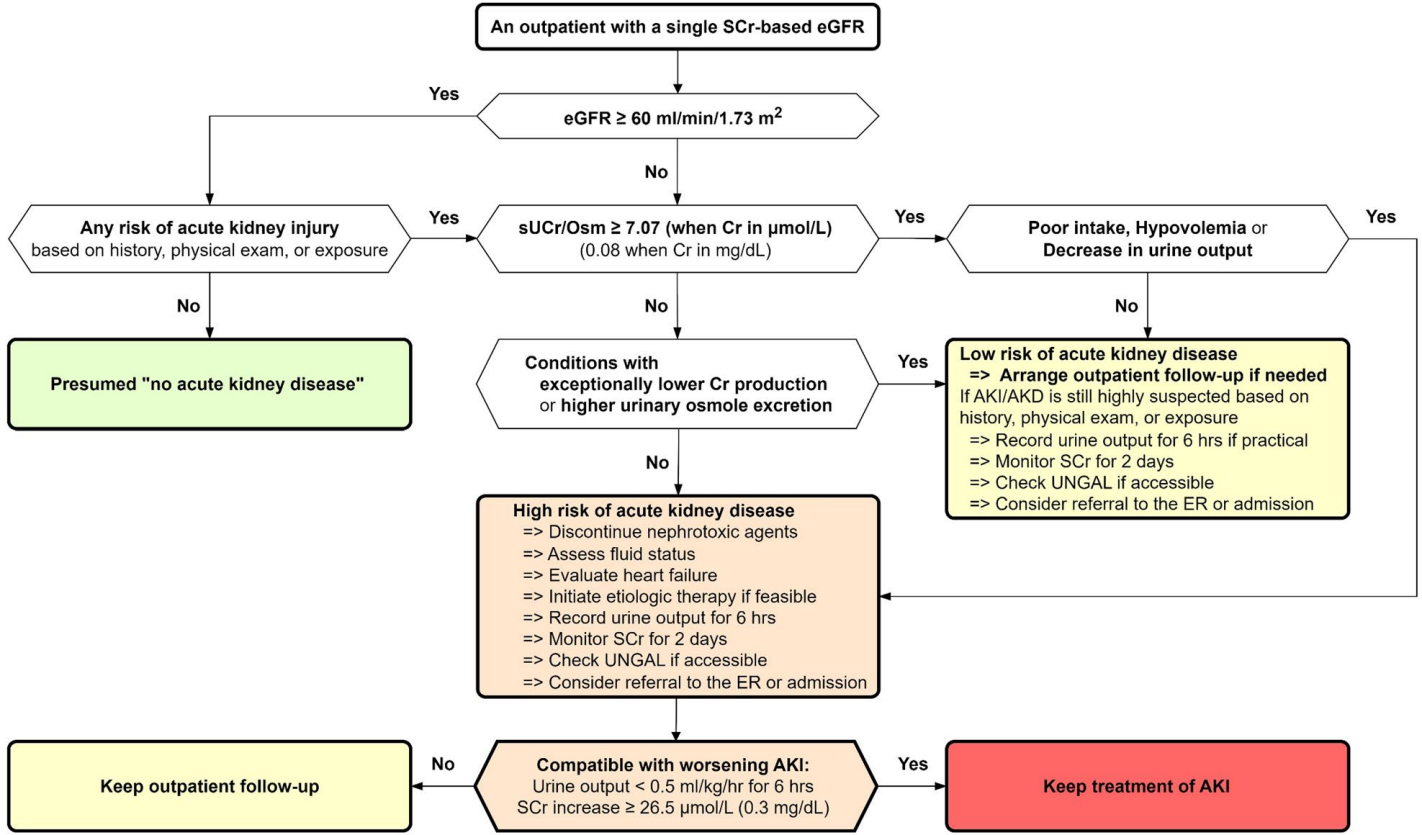
Flowchart for outpatient management based on a single serum creatinine (SCr)-based estimated glomerular filtration rate (eGFR) and a spot urine creatinine-to-osmolality ratio (sUCr/Osm). AKI, acute kidney injury. UNGAL, urinary neutrophil gelatinase-associated lipocalin.

Based on the efficacy of sUCr/Osm for identifying ‘No AKD’ in outpatients demonstrated in the current study, a flowchart for outpatient care using a single SCr and a simple sUCr/Osm test was developed (Figure 3). Individuals with an SCr-based eGFR ≥ 60 mL/min/1.73 m^2^ do not require further testing due to the low prevalence of ‘AKD’ (2.4%). Individuals with a suspicious history, physical findings, or risk exposure may require the management plan for outpatients with an eGFR < 60 mL/min/1.73 m^2^, which is a population with a lower prevalence of ‘No AKD’ (76.8%). Further decision-making can be based on a readily available sUCr/Osm test and consideration of false-positive or false-negative results, if available. For individuals with a low risk of ‘AKD’, routine nonurgent follow-up may be adequate. However, those suspected of having a high risk of ‘AKD’ require urgent assessment and management.

## Data Availability

The dataset utilized in this secondary analysis study was consolidated from the primary datasets accessible upon request from the National Institute of Diabetes and Digestive and Kidney Diseases (NIDDK) Central Repository (https://repository.niddk.nih.gov/home/).
